# A Case of Comorbid Myxoma and Chronic Lymphocytic Leukemia: Not Just a Coincidence?

**DOI:** 10.1155/2014/142746

**Published:** 2014-04-29

**Authors:** Heather Laird-Fick, Ashish Tiwari, Santhosshi Narayanan, Ying Qin, Deepthi Vodnala, Manisha Bhutani

**Affiliations:** ^1^Department of Medicine, Michigan State University College of Human Medicine, 788 Service Road Suite B301, East Lansing, MI 48824, USA; ^2^Good Shepherd Medical Center, 700 East Marshall Avenue, Longview, TX 75601, USA; ^3^Pathology Department, EW Sparrow Hospital, 1215 East Michigan Avenue, Lansing, MI 48909-7980, USA; ^4^St. John Hospital and Medical Center, VEP, 2nd Floor, Cath Lab, Internal Mailbox 110, 22101 Moross Road, Detroit, MI 48236, USA; ^5^Center for Cancer Research, National Cancer Institute, Lymphoid Malignancies Branch, 10 Center Drive 10/12N226, Bethesda, MD 20892, USA

## Abstract

*Background*. It is unclear why cardiac myxomas develop. We describe a case of comorbid myxoma and chronic lymphocytic leukemia (CLL) to offer insights into the tumor's pathophysiology. *Case*. A 56-year-old female with recurrent venous thromboembolism developed embolic stroke. Transesophageal echocardiogram showed a 1.7 × 1 cm sessile left atrial mass at the interatrial septum. Histopathology revealed myxoma with a B cell lymphocytic infiltrate suggestive of a low grade lymphoproliferative disorder. Bone marrow biopsy and flow cytometry of blood and the cardiac infiltrate supported the diagnosis of atypical CLL. She was followed clinically in the absence of symptoms, organ infiltration, or cytopenia. After eighteen months, she developed cervical and axillary lymphadenopathy. Biopsy confirmed B cell CLL/small lymphocytic lymphoma. She elected to undergo chemotherapy with fludarabine, cyclophosphamide, and rituximab, with clinical remission. *Conclusions*. The coexistence of two neoplastic processes may be coincidental, but the cumulative likelihood is estimated at 0.002 per billion people per year. A shared pathogenic mechanism is more likely. Possibilities include chronic inflammation, vascular endothelial growth factor A, shared genetic mutations, changes in posttranslational regulation, or alterations in other cellular signaling pathways. Additional studies could expand our current understanding of the molecular biology of both myxomas and CLL.

## 1. Introduction

Cardiac myxoma is a rare clinical entity of unclear etiology. Familial variants exist [1], but most myxomas are sporadic tumors that, in developed countries, occur in middle aged women. Specific genetic mutations have been identified in familial Carney complex, but they do not appear to play a role in sporadic cases [2]. Although the exact etiopathogenesis of myxomas is unclear, it has been hypothesized that an inflammatory trigger such as herpes simplex virus (HSV)-1 infection could lead to benign hyperproliferation of the spindle cells and formation of myxoma [3]. Others have implicated vascular endothelial growth factor (VEGF) A [4]. This communication describes a case of a woman with chronic lymphocytic leukemia (CLL) and atrial myxoma to potentially offer insights into the pathophysiology of this rare cardiac neoplasm.

## 2. Case Presentation

**Figure 1:**
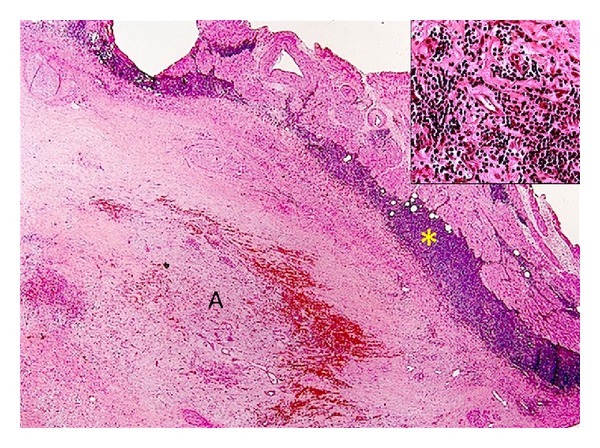
H&E stain. H&E stained slide demonstrating atrial myxoma tissue (A) and B cell lymphoma (*) and a 40x image (top left corner) showing B cell lymphocytic infiltrates into atrial myxoma.

A 56-year-old Caucasian female with a history of recurrent venous thromboembolism developed embolic stroke of the left pons and left occipital cortex while hospitalized with sepsis, pyelonephritis, and nephrolithiasis. Transesophageal echocardiogram showed a 1.7 × 1 cm sessile left atrial mass attached to the interatrial septum suggestive of myxoma, thrombus, or other tumor. The mass persisted at six weeks despite anticoagulation and antibiotic therapy. She therefore underwent surgical resection. Histopathological examination revealed myxoma with a B-cell lymphocytic infiltrate suggestive of a low grade lymphoproliferative disorder (Figure 1).

**Figure 2:**
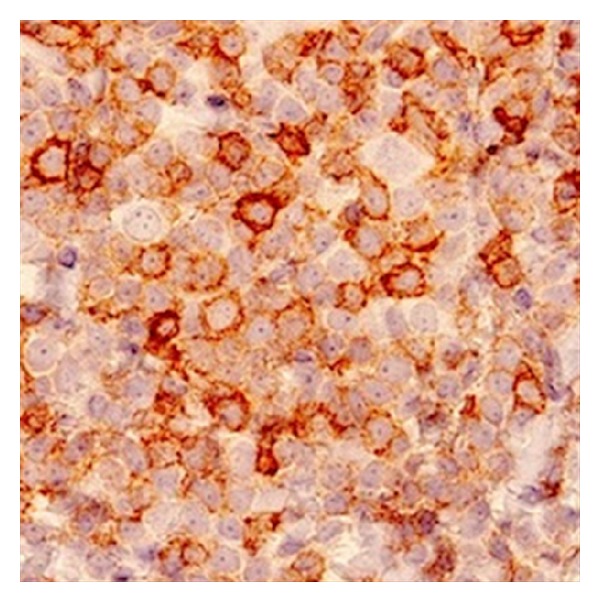
Positive immunohistochemical staining for CD5.

**Figure 3:**
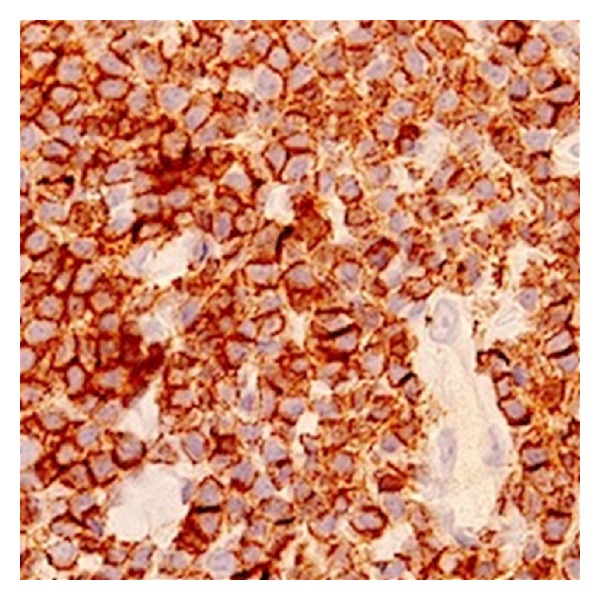
Positive immunohistochemical staining for CD20.

**Figure 4:**
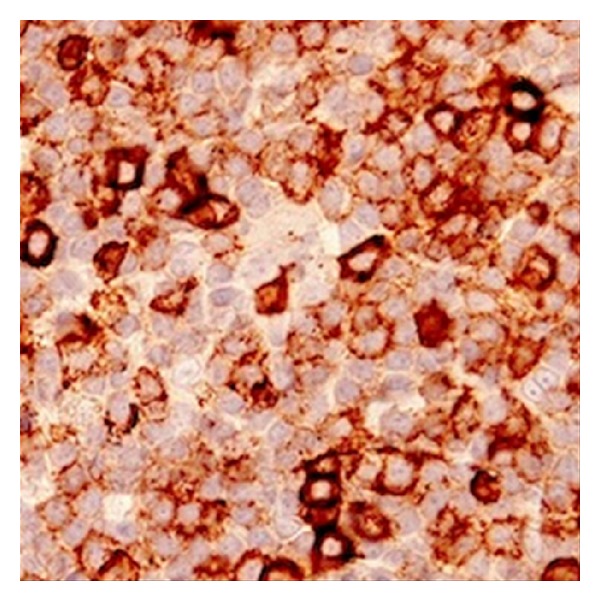
Positive immunohistochemical staining for CD43.

Further evaluation revealed a normal complete blood count. Peripheral smear showed 45% medium-sized lymphocytes with slightly irregular nuclei and small mature lymphocytes. Bone marrow biopsy was hypercellular with normal trilineage hematopoiesis and 20% nodular lymphocyte infiltrates. Immunophenotype of this lymphocyte population was positive for CD19, CD22, CD5, CD43 (weak), and BCL-2 and negative for CD23, cyclin D, and BCL-6. Samples of immunohistochemical stains for CD5, CD20, and CD43 are shown in Figures 2, 3, and 4, respectively. Fluorescent* in situ* hybridization (FISH) panel for chromosomal abnormalities was negative for t(11;14). Flow cytometry of her blood and cardiac infiltrate revealed cells with an immunophenotype profile similar to the bone marrow. Further staging workup with computed tomography of the chest, abdomen, and pelvis was negative for hepatosplenomegaly or lymphadenopathy. Based on CD20 and CD5 coexpression without CD23 expression, a differential diagnosis of atypical CLL, CD5 positive marginal zone lymphoma, or cyclin D1 negative mantle cell lymphoma was considered. A final diagnosis of atypical CLL was favored based on the lack of t(11;14) on FISH analysis.

She was followed clinically in the absence of symptoms, organ infiltration, or cytopenia. After eighteen months, she developed cervical and axillary lymphadenopathy. Biopsy confirmed B-cell CLL/small lymphocytic lymphoma (SLL). Repeated echocardiogram revealed a small mass along the atrial septum, compatible with scar tissue from myxoma resection versus local recurrence. She was elected to undergo chemotherapy with fludarabine, cyclophosphamide, and rituximab (FCR), with clinical remission.

## 3. Discussion

The existence of two neoplastic processes—cardiac myxoma and CLL/SLL—in this patient may be coincidental. However, the chances of this are vanishingly small. In an Irish study, myxoma occurred at a rate of 0.5 cases per million people per year [5], while a Dutch study showed that middle aged women developed CLL at a rate of 4 per 100,000 people per year [6]. The cumulative likelihood of developing these as independent processes is, therefore, estimated to be 0.002 per billion people per year. With the world population at 7 billion, the likelihood of a single such cooccurrence is less than 1.5% per year. Cases of hairy cell leukemia and lymphoma within resected cardiac myxomas have been reported as well [7, 8], supporting our hypothesis that a common pathogenic pathway led to the development of both the myxoma and CLL/SLL in this patient.

Chronic inflammation has been hypothesized to cause myxoma development, although studies of HSV-1 and other viruses have had conflicting results. Myxomas have been reported in a few patients after immunosuppression for solid organ transplant [9]. Inflammation also appears to be a trigger for CLL development [10]. Our patient was not profoundly immunosuppressed but had chronic inflammation related to her severe, recurrent deep venous thrombosis.

Vascular endothelial growth factor (VEGF) A plays a role in both myxoma and CLL [4, 11] but is unlikely to be causal in either. VEGF production normally increases with hypoxia and promotes angiogenesis and vascular permeability. CLL cells autonomously produce high levels of VEGF, which, in turn, may contribute to lymphocyte marginalization. Myxoma cells express VEGF receptors and form highly vascular structures. Benign lymphocytic infiltrates within myxomas have been described. In our patient, the CLL infiltrate likely created a VEGF-rich microenvironment, which in turn may have resulted in rapid growth of the cardiac tumor. Cases of thymoma [12], metastatic transitional cell carcinoma [13], and extramedullary hematopoiesis [14] within myxomas also suggest that myxomas provide a hospitable microenvironment. Yet, it seems unlikely that elevated VEGF alone triggered the proliferation of normal cardiac spindle cells.

A shared genetic defect is the more plausible explanation. Little is known about the genetic defects leading to myxomas. Myxomas have developed in children after chemotherapy, suggesting the role of acquired genetic mutations [15]. Abnormalities in chromosomes 12 and 17 have been reported in two cases [16]. Interestingly, CLL/SLL has also been linked to genetic defects on chromosomes 12 and 17, although in different regions than in myxoma, among others [17, 18]. Changes in specific microRNAs, important for posttranslational regulation, have been identified in CLL [19], but no similar work has been done in myxomas. The protein kinase-A (PKA) pathway has been implicated in both diseases as well, [20, 21] with specific PRKAR1A gene mutations reported in patients with the Carney complex [1]. Alternatively, an unknown genetic defect could be the precursor to both.

The synchronous development of two seemingly unrelated tumors in our patient raises interesting questions about the pathogenesis of cardiac myxomas. Additional studies to explore candidate genes, posttranslational regulation, or cellular signaling pathways could expand our current understanding of the molecular biology of both myxomas and CLL.
